# Malaria transmission after five years of vector control on Bioko Island, Equatorial Guinea

**DOI:** 10.1186/1756-3305-5-253

**Published:** 2012-11-12

**Authors:** Hans J Overgaard, Vamsi P Reddy, Simon Abaga, Abrahan Matias, Michael R Reddy, Vani Kulkarni, Christopher Schwabe, Luis Segura, Immo Kleinschmidt, Michel A Slotman

**Affiliations:** 1 Department of Mathematical Sciences and Technology, Norwegian University of Life Sciences, Ås, Norway; 2 Department of Entomology, Texas A&M University, College Station, TX, USA; 3 National Malaria Control Program, Ministry of Health, Malabo, Equatorial Guinea; 4 Medical Care Development International, Silver Spring, MD, USA; 5 Department of Epidemiology and Public Health, Yale University, New Haven, CT, USA; 6 Swiss Tropical and Public Health Institute, Basel, Switzerland; 7 MRC Tropical Epidemiology Group, Department of Infectious Disease Epidemiology, London School of Hygiene and Tropical Medicine, London, UK

**Keywords:** Entomological inoculation rate, Sporozoite, Human biting, Light traps, *Anopheles*

## Abstract

**Background:**

Malaria is endemic with year-round transmission on Bioko Island. The Bioko Island Malaria Control Project (BIMCP) started in 2004 with the aim to reduce malaria transmission and to ultimately eliminate malaria. While the project has been successful in reducing overall malaria morbidity and mortality, foci of high malaria transmission still persist on the island. Results from the 2009 entomological collections are reported here.

**Methods:**

Human landing collections (HLC) and light trap collections (LTC) were carried out on Bioko Island, Equatorial Guinea in 2009. The HLCs were performed in three locations every second month and LTCs were carried out in 10 locations every second week. Molecular analyses were performed to identify species, detect sporozoites, and identify potential insecticide resistance alleles.

**Results:**

The entomological inoculation rates (EIR) on Bioko Island ranged from 163 to 840, with the outdoor EIRs reaching > 900 infective mosquito bites per year. All three human landing collection sites on Bioko Island had an annual EIR exceeding the calculated African average of 121 infective bites per year. The highest recorded EIRs were in Punta Europa in northwestern Bioko Island with human biting rates of 92 and 66 mosquito landings per person per night, outdoors and indoors, respectively. Overall, the propensity for mosquito biting on the island was significantly higher outdoors than indoors (p < 0.001). Both *Anopheles gambiae* s.s. and *An. melas* were responsible for malaria transmission on the island, but with different geographical distribution patterns. Sporozoite rates were the highest in *An. gambiae* s.s. populations ranging from 3.1% in Punta Europa and 5.7% in Riaba in the southeast. Only the L1014F (*kdr*-west) insecticide resistance mutation was detected on the island with frequencies ranging from 22-88% in *An. gambiae* s.s. No insecticide resistance alleles were detected in the *An. melas* populations.

**Conclusions:**

In spite of five years of extensive malaria control and a generalized reduction in the force of transmission, parasite prevalence and child mortality, foci of very high transmission persist on Bioko Island, particularly in the northwestern Punta Europa area. This area is favorable for anopheline mosquito breeding; human biting rates are high, and the EIRs are among the highest ever recorded. Both vector species collected in the study have a propensity to bite outdoors more frequently than indoors. Despite current vector control efforts mosquito densities remain high in such foci of high malaria transmission. To further reduce transmission, indoor residual spraying (IRS) needs to be supplemented with additional vector control interventions.

## Background

The Bioko Island Malaria Control Project (BIMCP) in Equatorial Guinea is a public-private and civil-society partnership between Marathon Oil Corporation and its corporate partners; Medical Care Development International, a private voluntary organization; academic institutions; and the Government of Equatorial Guinea. The first five year phase of the project began in 2004 and was extended by a second five year term starting in 2009. The goal of the project is to substantially reduce malaria transmission and its associated morbidity and mortality and ultimately to potentially eliminate malaria on Bioko Island. The malaria control strategy of the BIMCP is based on a set of integrated interventions combining vector control, effective case management, prevention of malaria during pregnancy, behavioral change communications, monitoring and evaluation, and operational research [1]. The mosquito vector suppression activities include twice-yearly indoor residual spraying (IRS) of insecticides on interior walls of all inhabited dwellings. In 2007 long-lasting insecticide treated bed-nets (LLIN) were distributed to all households to cover all sleeping areas [2].

Case management consists of improved case detection and access to artemisinin combination therapy to treat diagnosed cases. Pregnant women are provided with intermittent preventive treatment with two doses of sulfadoxine-pyrimethamine(SP), at least 30 days apart and LLINs at antenatal care visits, and confirmed cases are treated with quinine and given iron supplementation. An integrated behavioral change and communication (BCC) and information, education and communication (IEC) component supports project activities. In addition, a comprehensive island-wide monitoring and evaluation program has been established, which includes surveillance of malaria cases, vector monitoring, assessment of transmission intensity, insecticide efficacy and resistance, insecticide and medicine stocks, and support to improve the national health information system.

The results of these concerted efforts reduced malaria prevalence from 42% to 18% in children two to five years old and all cause under-five mortality from 155 per 1000 births to 52 per 1000 between 2004 and 2008 [3]. Although there was substantial variation in child malaria between sentinel sites, significant reductions were achieved in most sites [3] with the exception of the northwestern coastal region of Punta Europa, where malaria transmission appeared to be largely unaffected and remained at high levels [3-5].

During 2004–2008, vector monitoring was conducted through a household-based network of window exit traps installed in six houses in each of sixteen sentinel sites. The abundance of *An. melas* and *An. funestus* collected in window traps after the first spray round were reduced by 87% and 85%, respectively, suggesting high susceptibility to pyrethroids. The number of *An. funestus* collected continued to decline, and only three specimens were found after the third spray round [6]. The abundance of collected *Anopheles gambiae* s.s. appeared to be less affected by the first spray round with pyrethroids, but did decline by 93% after introduction of bendiocarb during the second spray round. Despite this apparent lack of impact on *Anopheles gambiae* densities, the first spray round did have a significant impact on transmission [1,6], probably due to the reduction in *An. gambiae* sporozoite rates after the spray round, which could have resulted from reduced longevity due to spraying.

Both M and S molecular forms of *An. gambiae* s.s. were identified. The *kdr* resistance mutation was detected with a high frequency in the M form at Punta Europa (85%) [6], but was also present in the S form (Kleinschmidt, unpublished data). Subsequently, the proportion of *An. gambiae* that belonged to the S molecular form declined rapidly [6]. The window trap data suggested that the densities of malaria vectors on Bioko Island were reduced to very low levels during the first phase of the project. After the third spray round, only 139 mosquitoes were collected in the window traps, and none of these tested positive for *Plasmodium* sporozoites [6]. However, this apparently low mosquito abundance did not correspond with data from parasitemia surveys showing high malaria prevalence in certain areas where few mosquitoes were collected.

Though there is evidence in areas with intense transmission, like Bioko, that reductions in parasite prevalence can lag substantially behind reductions in entomological indicators of the effects of vector control [7], this evidence called into question the quality of the vector monitoring system being employed on Bioko Island. In 2008 a review of the performance of window traps revealed that the traps were functioning sub-optimally and that light trap collections (LTC) and human landing collections (HLC) collected a much larger number of mosquitoes. This resulted in a decision to fundamentally change the vector monitoring strategy. This new strategy focused on adult mosquito collections through HLC and LTC to monitor vector density, species composition, insecticide resistance allele frequencies and sporozoite rates. In addition, operations research with the aim of answering critical emerging research questions were incorporated in the BIMCP [8-11]. Here we report the results from the vector monitoring conducted in 2009, based on this new strategy and describe vector density, biting times and venue, species composition, sporozoite rates, entomological inoculation rates (EIR), and frequency of insecticide resistance alleles.

## Methods

### Study area

Bioko Island belongs to Equatorial Guinea and is located in the Bay of Guinea in Central Africa about 30 km southwest of the Cameroon coast. The surface area of Bioko Island is ~2,000 km^2^. Most of the island’s 260,000 inhabitants live in the northern part of the island. The discovery of large offshore oil reserves in the 1990’s has resulted in rapid economic growth and an overall increase in incomes, although a substantial proportion of the island’s residents still practice subsistence agriculture and fishing. The interior of the island is covered with dense forests on the steep slopes of volcanoes and calderas. The highest peak on the island reaches 3,011 m above sea level. The mean annual rainfall is ~2,000 mm/year. The rainy season starts in May and ends in October with peaks in August and September of ~300 mm/month. Mean daily maximum and minimum temperatures range between 29-32°C and 19-22°C, respectively. The island has a humid tropical environment. Malaria is endemic with year-round transmission. A set of 18 sentinel sites were established in 2004 as part of the BIMCP to monitor entomological, clinical and population indicators [1].

### Mosquito collections

**Figure 1:**
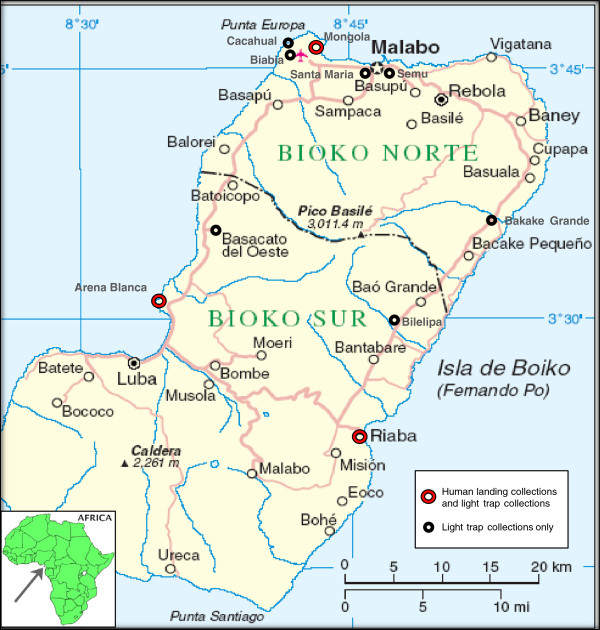
**Mosquito collection sites of the Bioko Island Malaria Control Project, Bioko Island, Equatorial Guinea.** The three sites in the Punta Europa area together make up the Punta Europa sentinel site. Arena Blanca is not part of the original sentinel sites, but was added as a mosquito collection site in 2009. (Source: Map No. 3861 Rev. 4, January 2005, Department of Peacekeeping Operations Cartographic Section, United Nations. http://www.un.org/Depts/Cartographic/map/profile/eqguinea.pdf.).

Mosquitoes were collected using a combination of HLC conducted every two months and LTC performed approximately every two weeks. The HLC were carried out to determine reliable estimates of the human biting rate. Ethical clearance for the use of HLC was obtained from the National Malaria Control Program of the Ministry of Health and Social Welfare of the Equatoguinean government. HLC were carried out in three sites; Mongola (northwestern Punta Europa region), Arena Blanca (southwestern Luba region), and Riaba (southeastern region); in March, May, July, September, and November 2009 (Figure 1). These sites were selected based on being as representative as possible for the island and still logistically feasible to carry out, but also for the importance of monitoring mosquito populations in areas with a high number of malaria cases, such as in the Punta Europa area. Arena Blanca is a new collection site which was added in 2009 and was not part of the original sentinel sites in which window trap collections were performed. Collections were carried out during two consecutive days in each collection month, except in March in Mongola, when four days of consecutive collections were performed. In each site, four houses were randomly selected from a list of houses used in the annual BIMCP parasitemia survey. Where feasible, the same houses were used throughout the year. In each house, two local volunteers, one positioned indoors and the other outdoors, collected mosquitoes landing on exposed legs and feet from 19:00 to 06:00. Volunteers were informed about the potential risks of collecting mosquitoes, gave informed consent in writing, and were offered treatment if they became ill. The collected mosquito specimens were combined per collection hour. Collectors changed between indoor and outdoor positions at midnight. For each site eight people performed collections per night (4 outdoors and 4 indoors), resulting in a total of 128 indoor and 128 outdoor collection nights during 2009. In addition to these HLCs, indoor LTCs were carried out in the same houses and during the same nights in the same way as described below.

Regular indoor LTCs were carried out to estimate seasonal and spatial variations in indoor mosquito density. These were performed in 10 sites around the island (Figure 1) on an approximately biweekly basis from February to December 2009. These sites were selected based on local malaria transmission (malaria prevalence stratified into five categories derived from annual parasitemia surveys [4] and unpublished data), geographical location, and whether sites were urban or rural (Table 1). Seven sentinel sites coincided with locations from which window trap data are available [6]. Two additional sites (Biabia and Cacahual) were selected in Punta Europa to strengthen monitoring in this area of high malaria prevalence and the tenth site was Arena Blanca. A modified CDC light trap with ultraviolet light emitting diode (UV LED) [12] was installed in an occupied bedroom, about 1.5 m above the floor, 20–50 cm from a mosquito net (if present) and/or ca. 50–100 cm from the nearest window to the bed. Rain shields were removed from traps so as not to obstruct the light source unduly or interfere with the daily life of household inhabitants.

**Table 1:** Selected sentinel sites of the Bioko Island Malaria Control Project, Equatorial Guinea for vector monitoring

**Sentinel site**	**Malaria prevalence (%)**	**Geographical location**	**Setting**	**Collection method**
1. Punta Europa – Mongola	> 50	North West	Rural	HLC + LTC
2. Punta Europa - Biabia	> 50	North West	Rural	LTC
3. Punta Europa - Cacahual	> 50	North West	Rural	LTC
4. Riaba	30-40	South East	Rural	HLC + LTC
5. Basacato del Oeste	30-40	South East	Rural	LTC
6. Santa Maria	30-40	North	Urban	LTC
7. Arena Blanca	30-40	South West	Rural	HLC + LTC
8 Bilelipa	20-30	East	Rural	LTC
9. Semu	10-20	North	Urban	LTC
10. Bakake Grande	< 10	East	Rural	LTC

Selection was based on combined malaria prevalence during 2004–2008 (from Kleinschmidt *et al.*[4] and unpublished data), geographical location, and setting. HLC = Human Landing collections. LTC = Light trap collections.

Where possible, traps were placed in the same location within the home for each sampling event. Light traps were operating from 18:00 to 06:00 during one night in five houses per site in the biweekly LTC and during two nights in each of four houses per site in the bimonthly LTC. To summarize, there were seventeen LTC events in all ten sites and an additional five LTC events coinciding with the HLC, totalling 938 LTC trap-nights.

### Precipitation

Rainfall data were collected daily from Mongola during the whole study period using a standard plastic rain gauge.

### Specimen handling and molecular analyses

Adult specimens were brought back to the laboratory where they were sorted, counted, and classified according to genus and blood feeding status (unfed or fed) during each collection event. Specimens, separated by collection method, day, and hour were placed in 70% alcohol and stored until shipped to Texas A&M University for molecular analysis. Heads and thoraces of non-bloodfed mosquitoes were dissected and subjected to DNA extraction using a QIAGEN DNeasy Blood & Tissue Kit on a QIAGEN Biosprint (QIAGEN Sciences Inc., Germantown, MD). A diagnostic PCR followed by restriction enzyme digest was used for species identification within the *An. gambiae* s.l. complex, as well as molecular form [13,14]. *Plasmodium falciparum* sporozoite detection was performed using the Pf1 and Pf2 primers developed by Morassin *et al*. [15] using slightly modified PCR conditions. Detection of the knock-down resistance alleles L1014F and L1014S (previously referred to as *kdr-*west and *kdr-*east, respectively), as well as the insensitive acetylcholinesterase (*iAChE*) allele was performed using the real-time Taqman assays developed by Bass *et al.*[16,17].

### Data analyses

The Wilcoxon signed-rank test was conducted to evaluate whether more mosquitoes were collected outdoors than indoors by HLC. The total number of mosquitoes collected outdoors and indoors for each collection day and location (n=32 collection events) were used in the analysis. The test was carried out in IBM SPSS Statistics 19. The sporozoite rates between *An. melas* and *An. gambiae* s.s. in the LTC in Arena Blanca were compared by the two-proportion Z-test using a web based tool ( http://in-silico.net/tools/statistics/ztest/).

## Results

### Human landing collections (HLC)

**Figure 2:**
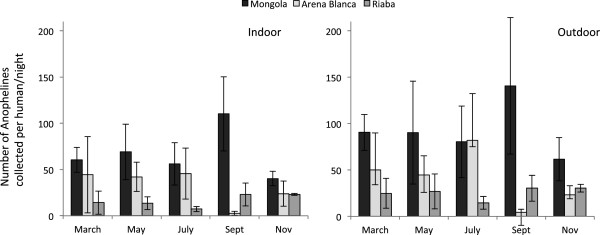
**Anopheline mosquito density on Bioko Island, Equatorial Guinea in 2009.** Number of anopheline mosquitoes collected by human landing collections indoors (left panel) and outdoors (right panel) per person per night in three locations. The dominant species in Mongola was *An. gambiae* s.s.; in Arena Blanca *An. melas*; and in Riaba both species were almost equally abundant. Bars indicate 95% confidence intervals.

**Figure 3:**
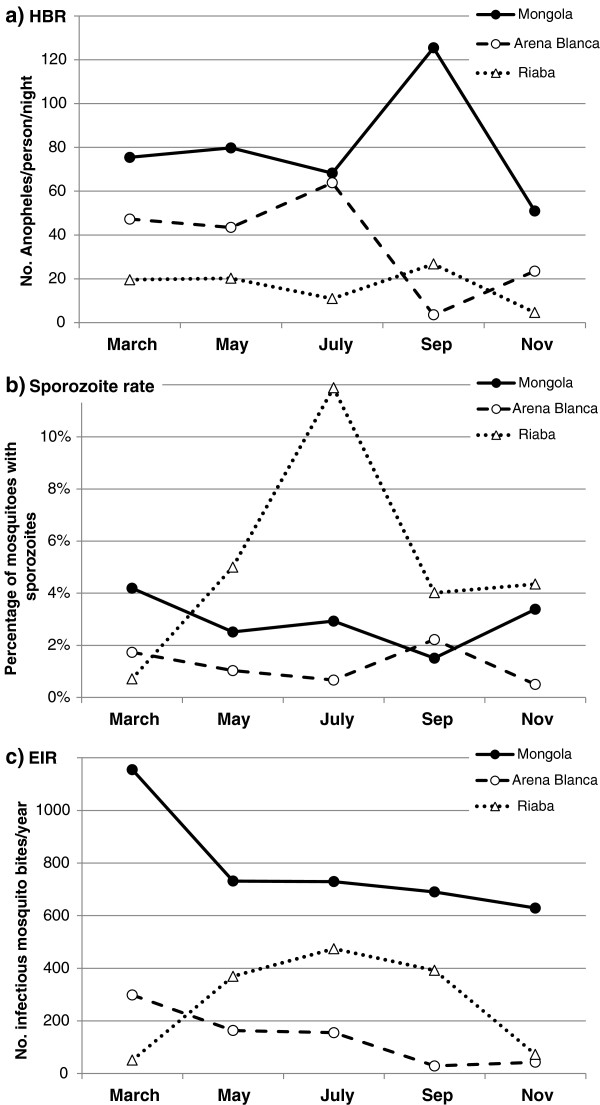
**Entomological transmission parameters on Bioko Island, Equatorial Guinea in 2009.** Each monthly mosquito collection event was used to calculate **a**) human biting rates (HBR), **b**) sporozoite rates, and **c**) entomological inoculation rates (EIR).

A total of 11,822 *Anopheles* mosquitoes were collected by HLC in the three locations, of which 3,043 were further analyzed (Table 2). Mongola, one of the villages in the Punta Europa area in the northwest part of the island, had by far the highest human biting rate (HBR) averaging 79.2 mosquitoes per person night, yielding 7,604 (4,432 outdoors vs. 3,172 indoors), which represented 64.3% of the total number of mosquitoes collected by HLC on Bioko Island in 2009. In Arena Blanca and Riaba, the average HBRs were 36.3 and 16.4 respectively, resulting in 2,905 collected specimens in Arena Blanca (1,635 outdoors vs. 1,270 indoors) and 1,313 mosquitoes in Riaba (816 outdoors vs. 497 indoors). The number of anopheline mosquitoes collected throughout 2009 in the HLC did not show a consistent pattern across the three sites (Figure 2, Figure 3a). For example, in Mongola the number of mosquitoes was high in September, both indoors and outdoors, whereas in Arena Blanca few mosquitoes were collected in this month. In Riaba, relatively little variation in the number of mosquitoes collected was observed throughout the year.

**Table 2:** Number of anophelines collected and analyzed for species composition, and average sporozoite rates in human landing collections

**Location**	**No. *Anopheles *collected (no. analyzed)**	**Species composition**				***Pf *sporozoite rate% (± 95% C.I.)**	
		***An. gambiae *s.s.**		***An. melas***		***An. gambiae *s.s.**	***An. melas***
Mongola	7,604 (1,211)	1,207	99.6%	4	0.3%	3.1 (± 1.0)	0
Arena Blanca	2,905 (917)	71	7.7%	846	92.3%	0	1.2 (± 0.7)
Riaba	1,313 (915)	423	46.2%	492	53.8%	5.7 (± 2.2)	2.8 (± 1.5)

Mosquitoes collected both indoors and outdoors in 2009 in Mongola, Arena Blanca, and Riaba, Bioko Island, Equatorial Guinea. Sporozoite rates averaged over the whole year. *Pf*=*Plasmodium falciparum*. C.I.=Confidence intervals.

The Wilcoxon test showed that, overall for the island, significantly more *Anopheles* mosquitoes were collected outdoors than indoors by human landing (Z = −3.626, n = 32, p < 0.001). Specifically, significantly more mosquitoes bit human subjects seated outdoors than indoors in Mongola (Z = −2.59, p = 0.01, n = 12) and Riaba (Z = −2.701, p = 0.007, n = 10), but not in Arena Blanca (Z = −1.481, p = 0.139, n = 10).

The three sites represent different species composition profiles, with Mongola collections consisting almost exclusively of *An. gambiae* s.s. (99.6%). In contrast, the collections from Arena Blanca consisted of 92.3% *An. melas* with the remainder being *An. gambiae* s.s*.* Both species were almost equally abundant in Riaba, with 46.2% belonging to *An. gambiae* s.s. and 53.7% to *An. melas.* Every *An. gambiae* s.s. identified from the HLC belonged to the M molecular form. For 62 mosquitoes the species diagnostic PCR did not yield a result. At least some of these failures were due to technical error as the *An. gambiae* s.l. specific Taqman assays to detect *kdr* and/or *ace-1*^R^ worked for 35 of these. However, we cannot rule out the possibility that less than 1% of collected samples represent other anopheline species that were known to be present on Bioko Island [18].

**Figure 4:**
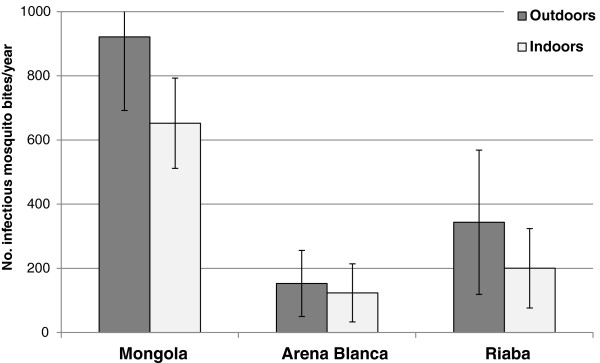
**Outdoor and indoor entomological inoculation rates (EIR) in three sites on Bioko Island, Equatorial Guinea in 2009.** Bars indicate 95% confidence intervals.

The HBRs and sporozoite rates varied over the year with different patterns across sites. This produced large variations in EIR across sites and seasons (Figure 3). In Mongola the EIR was high in the beginning of the year and then steadily decreased; a pattern that was also observed in Arena Blanca, although the magnitude was much lower. In Riaba there was a peak in EIR in the middle of the year, caused by the very high sporozoite rates in July. The overall EIR in Mongola was calculated to be 840 infective mosquito bites per year, with outdoor and indoor EIRs being 922 and 652, respectively (Figure 4). In Riaba the overall EIR was 311 (EIR_outdoor_=344; EIR_indoor_=200) and in Arena Blanca 163 (EIR_outdoor_=153; EIR_indoor_=123). *Anopheles gambiae* s.s. and *An. melas* contributed 100% to the respective EIRs in Mongola and Arena Blanca. In Riaba, *An. gambiae* s.s. and *An. melas* contributed approximately 60% and 40%, respectively to the EIR.

### Biting times and venue

**Figure 5:**
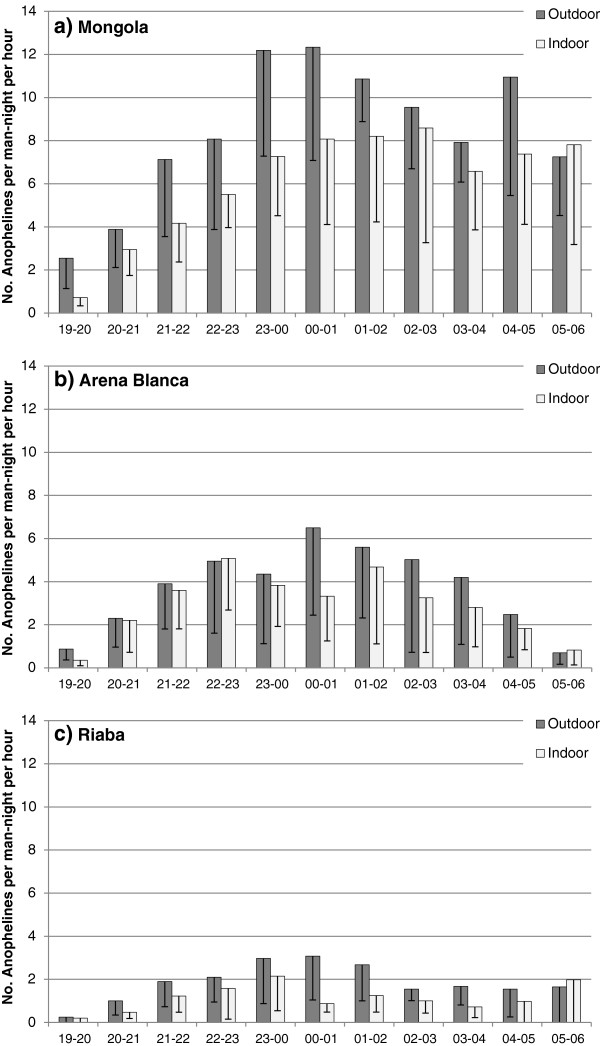
**Human landing collections per hour on Bioko Island, Equatorial Guinea.** Anopheline mosquitoes collected outdoors and indoors by human landing collections in **a**) Mongola, **b**) Arena Blanca, and **c**) Riaba, Bioko Island, 2009. Bars indicate 95% confidence intervals.

In all locations, the majority of the biting took place outdoors throughout the night (Figure 5). The biting rates of *An. gambiae* s.s. in Mongola presented here have been reported before [8]. Biting activity increased steadily until midnight and was high throughout the rest of the night. In Arena Blanca, *Anopheles* biting rates, representing mostly *An. melas,* peaked around midnight, although biting activity continued throughout the night. The hourly mosquito biting rates in Riaba, although lower, showed a similar pattern as in Arena Blanca. In all three locations, a substantial amount of the biting activity occurred before midnight, when many people are active outdoors and not protected by IRS and/or LLINs [8]. In Mongola, the proportion of *Anopheles* mosquitoes collected before midnight outdoors was 35% and indoors 30%. The corresponding proportions for Arena Blanca were 40% outdoors and 47% indoors; and for Riaba 40% outdoors and 45% indoors.

### Light trap collections (LTC)

**Figure 6:**
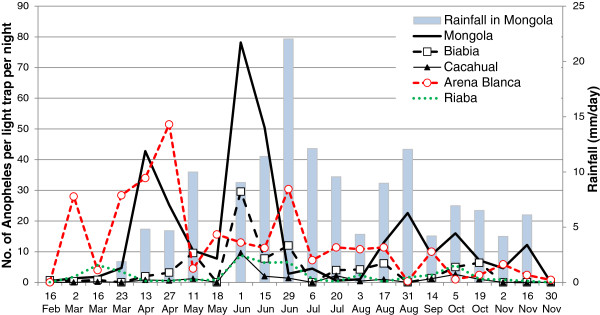
**Anophelines collected in light traps and daily precipitation in Mongola, Equatorial Guinea.** Bi-weekly light trap collections in five of ten sites and amount of daily rainfall in Mongola, Punta Europa, 2009, Bioko Island, Equatorial Guinea. Densities in Basacato del Oeste, Bilelipa, Bakake Grande, Semu, and Santa Maria never exceeded 1.2 anophelines per light trap per night (not shown). Mosquito collections were carried out on the dates on the x-axis. The rainfall bars indicate the average amount of rainfall per day during the period previous to each mosquito collection date.

The light trap collections yielded far fewer mosquitoes than the HLC. A total of 4,083 *Anopheles* mosquitoes were collected in ten locations during a total of 938 LTC nights. There were large temporal and spatial variations in the number of mosquitoes collected throughout the year (Figure 6). Five sites (Mongola, Arena Blanca, Biabia, Riaba, and Cacahual) together produced > 98% of all *Anopheles* mosquitoes collected by LTC on Bioko in 2009 (Table 3). The density of *Anopheles* mosquitoes collected per trap-night was also highest in these same sites.

**Table 3:** Entomological data from light trap collections in 2009 on Bioko Island, Equatorial Guinea

					***An. gambiae *s.s.**			***An. melas***		
**Site**	**No. collected(%)**	**No. collection events**	**Total no. trap nights/site**	**Density**	**Proportion**	**L1014F**	***Pf *Sporozoite rate**	**Proportion**	**L1014F**	***Pf *Sporozoite rate**
Mongola	1754 (43.0)	22	134	14.4 (±8.2)	99.7 (±0.6)	50.6 (±3.7)	1.4 (±1.3)	0.3 (±0.6)	0	0
Biabia	470 (11.5)	17	90	5.5 (±3.4)	99.0 (±1.1)	43.2 (±4.0)	0.7 (±0.9)	1.0 (±1.1)	0	33.3 (±53.3)
Cacahual	121 (3.0)	17	89	1.4 (±1.1)	87.3 (±7.7)	30.8 (±7.9)	8.1 (±6.8)	12.7 (±7.7)	0	0
Basacato del Oeste	0 (0.0)	14	64	0	-	-	-	-	-	-
Arena Blanca	1408 (34.5)	21	111	13.5 (±8.2)	11.4 (±2.6)	43.1 (±8.5)	1.5 (±3.0)	88.6 (±2.6)	0	1.0 (±0.9)
Riaba	285 (7.0)	21	125	2.4 (±1.1)	18.6 (±8.4)	22.2 (±13.6)	0	81.4 (±8.4)	0	4.3 (±4.7)
Bilelipa	1 (0.02)	16	85	0.01 (±0.02)	0	0	0	100	0	0
Bakake Grande	9 (0.2)	15	80	0.1 (±0.2)	100	42.9 (±25.9)	0	0	0	0
Semu	24 (0.6)	16	80	0.3 (±0.2)	100	87.5 (±11.5)	6.3 (±11.9)	0	0	0
Santa Maria	11 (0.3)	16	80	0.1 (±0.1)	100	66.7 (±26.7)	16.7 (±29.8)	0	0	0
TOTAL / Average	4083 (100.0)	175	938	4.7 (±1.6)	58.0 (±2.6)	46.0 (±3.4)	1.8 (±0.9)	62.0 (±2.6)	0	1.7 (±0.6)

Total number, density per light-trap night, proportion of *An. gambiae* s.s. and *An. melas* collected in 10 sites, frequency of knockdown resistance mutation L1014F (*kdr*-west) (%), and *Plasmodium falciparum* (*Pf*) sporozoite rates (%) found in each species (95% confidence intervals in parentheses).

The three Punta Europa sites (Mongola, Biabia, and Cacahual) comprised 57.5% of all mosquitoes, with the majority in Mongola (1,754 mosquitoes, 43% of the total). In Arena Blanca and Riaba light traps collected 1,408 (34.5%) and 285 (7.0%) mosquitoes, respectively. In many locations the light traps collected very few mosquitoes such as in Basacato del Oeste and Bilelipa (Table 3). In six of the ten sites, *An. gambiae* s.s. was the most common mosquito collected whilst *An. melas* was the most abundant vector only in Arena Blanca and Riaba (Table 3). All *An. gambiae* s.s. belonged to the M molecular form.

In the Punta Europa area (Mongola, Biabia, and Cacahual) the number of anopheline captures in light traps peaked during the 1^st^ week of June, whereas in Arena Blanca the main peak occurred during late April. For the Punta Europa sites it seems that reduced mosquito captures generally followed peaks in precipitation, indicating that very high levels of rainfall can reduce the larval productivity.

Sporozoite rates in the LTC could be estimated from only a few locations with any degree of accuracy (Table 3), as the number of collected mosquitoes was low in many locations for both *An. gambiae* s.s. and *An. melas,* i.e. most of the confidence interval estimates included 0%. The sporozoite rates for *An. gambiae* s.s. were 1.4% (± 1.3) in Mongola and 8.1% (± 6.8) in Cacahual. The overall sporozoite rate for all the *An. gambiae* s.s. LTC captures was 1.8% (± 0.9). The sporozoite rate in *An. melas* from the LTC in Arena Blanca was estimated at 1.0% (±0.9), which was not significantly different from that in *An. gambiae* in LTC in this location (z = 0.6251, p = 0.56).

### Insecticide resistance

Both *An. gambiae* and *An. melas* populations were screened for the presence of three insecticide resistance alleles; L1014F (*kdr*-west) and L1014S (*kdr*-east)*,* which provide resistance to DDT and pyrethroids and *ace-1*^*R*^, which provides resistance against carbamates. The only insecticide mutation found was the L1014F and it was only present in *An. gambiae* s.s. The frequency of this allele was quite heterogeneous across the island ranging from 22.2% to 87.5%, averaging 48.3% across eight populations in the LTC. The highest frequencies observed in the urban areas of Semu and Santa Maria (Table 3). The L1014F frequencies obtained in the HLC ranged from 16.9% to 51.3% (Table 4). No resistance alleles were detected in the *An. melas* populations.

**Table 4:** **Insecticide resistance in *An. gambiae *s.s. (M form) captured by HLC on Bioko Island, Equatorial Guinea**

			***kdr *genotypes**			
**Location**	**L1014F frequency**	**n**	**S/S**	**S/R**	**R/R**	**HWD**
Mongola	51.3% (± 2.0)	1195	297	570	328	p = 0.29
Arena Blanca	39.0% (± 5.8)	136	48	70	18	p = 0.64
Riaba	16.9% (± 2.5)	423	300	103	20	p = 0.024

S = L1014L, the susceptible allele. R = L1014F, the resistant allele.

Frequency of L1014F resistance mutation (*kdr*-west); numbers of mosquitoes tested (n) and identified with susceptible and resistant alleles; and p-values for chi-square test of Hardy Weinberg disequilibrium (HWD) from mosquito samples collected by human landing in 2009 in three locations.

### Precipitation

The rainy season in 2009 started in March and ended in November (Figure 6). The total amount of rainfall was 1920 mm with the two major peaks in June (510 mm) and August (335 mm). More than half of the yearly rainfall occurred in June-August. These months also had more than 60% rainy days per month. A single tropical storm on 22 June produced 178 mm in one day.

## Discussion

The results presented here show that, after five years of malaria vector control interventions, and in spite of reductions in the prevalence of human malaria on Bioko Island [1,3], foci of persistent intensive malaria transmission remained. High human biting and sporozoite rates in Punta Europa, in the north-western part of the island, indicate that the human population in this area are potentially exposed to about 840 infective mosquito bites per year when indoor and outdoor biting is taken into account. The outdoor annual EIR was 922 infective mosquito bites per year; using the data from the March collections only the EIR was close to 1200. These rates are among the highest entomological inoculation rates published in the literature [19-21]. An annual EIR of 1,235 infective bites per person was reported in the early 1990’s in Sierra Leone [22], although this value was later revised to 884 by Hay *et al.*[19]. All three sites where HLC were conducted in the present study had an annual EIR exceeding the calculated African average of 121 infective bites per year [19]. An EIR of 1,030 was reported from Riaba on Bioko Island in the late 1990s [23], suggesting that Bioko Island had one of the (if not the) highest rates of malaria transmission when the control efforts started, and in spite of the intensive and extensive nature of these control efforts, transmission still persists, indicating the need for sustained and scaled up control. This underscores how important it is to have a long-term control framework in place, one that can sustain control over more than the typical project cycle, and one that can adapt to changing transmission realities.

The high EIR in Punta Europa in 2009 resulted from both extremely high HBR and high sporozoite rates. In a review by Hay *et al*. [19] similar or higher HBRs than this were recorded only from Burundi [24] and Cameroon [25]. More recently, rainy season HBRs in Dakar, Senegal were recorded to be almost three times higher than those reported in the current study [26]. The Punta Europa area is unique for Bioko Island in that it is a fast-growing industrial and commercial zone, where the country’s international airport is located. The area is unusually flat compared to the rest of Bioko Island; it has a low forest cover, relatively poor drainage, and consists of many construction sites, all of which contribute to providing excellent mosquito breeding habitats.

Mosquito density in the Punta Europa area has a complex relationship with the daily amount and intensity of rainfall. For example, the 2009 LTCs showed that the high mosquito density in early June followed high rainfall in early May, supplemented by moderate rainfall in the second half of May (Figure 6). On the other hand, the sharp decline in mosquitoes on June 29 was likely due to one single heavy storm downpour on June 22 (included in the June 29 precipitation bar in Figure 6), which presumably flushed larvae out of their breeding habitats. The mosquito densities in Arena Blanca and Riaba were not affected by precipitation in Punta Europa, probably because of a different rainfall pattern in those locations. It could also be due to the higher number of *An. melas* collected in these locations. *Anopheles melas* might be affected differently than *An. gambiae* s.s. by rainfall patterns, although this was not studied here.

The *An. gambiae* s.s. populations in Punta Europa also have a relatively high sporozoite rate averaging ~3% and ranging between 1.0% (± 1.7% 95% CI) and 3.9% (± 1.9% 95% CI) over the year (data by month not shown). In Riaba the sporozoite rate in this species was much higher, at least in the HLC, but a much lower human biting rate resulted in a considerably lower EIR in this location.

Malaria parasites in Punta Europa are highly prevalent in the human population [4,5]. Although the high EIR values reported here indicate a high potential for transmission, this is only realized if the human population is not protected by effective control measures. We have previously reported high outdoor biting rates in this area [8], and given the high effective coverage with IRS and LLINs, a large fraction of the transmission between humans and mosquitoes probably occurs at outdoor venues, including bars and restaurants. Based on the data presented here outdoor biting seems to be prevalent throughout the island. The potential for malaria transmission is further aggravated by the observation that host seeking begins early in the evening. In the current HLCs, about 30-50% of indoor biting occurred before midnight when the protection from vector control measures is not optimal. About 35-40% of outdoor biting during HLC occurred before midnight, at a time when most people are active outside their houses and thus being greatly exposed to malaria mosquito bites. The potential for transmission in outdoor venues where people are not protected by IRS and LLIN is therefore considerable and underscores the need for the development of control methods that effectively target outdoor feeding populations in addition to IRS and LLINs.

A recent study from Bioko concluded that there is currently no epidemiological evidence of outdoor biting having an impact on malaria prevalence in children in Bioko [27]. Although the study found that spending time outdoors at night was rare amongst children under 15 years, those who did spend the previous night outdoors did not have a higher prevalence of infection than those who stayed indoors [27]. However, the power of that study to detect an impact of spending time outdoors was limited by the fact that few children slept under a bed net, and that the last IRS spray round was conducted 3–5 months prior to the survey, providing little protective effect to being indoors. Furthermore, adults are far more likely to stay outdoors at night than children and thereby potentially keeping parasites circulating in the general population. Further research is, therefore, required to determine the epidemiological impact of being outdoors in the evening and at night in both children and adults.

In 1998–1999, well before the implementation of the current control program, an annual EIR of 1,030 was reported for Riaba [23]. *Anopheles funestus* was the main contributor (76%) to this EIR, whereas *An. gambiae* was responsible for the remaining 24%. It is quite clear that the entomological situation has changed drastically in Riaba in the thirteen years since the first report. The present results indicate that *An. funestus* was absent or, at most, occurring at extremely low densities in Bioko, including Riaba. Presumably, the continuous BIMCP vector control activities, particularly IRS, have nearly eliminated *An. funestus* on Bioko; something which was also observed following the first IRS rounds [6]. Sporozoite rates are still high in Riaba among *An. gambiae* s.s. and *An. melas*, indicating that parasite transmission from human to mosquito is high in the area, despite relatively low biting rates compared to the rest of the island. The extreme peak in sporozoite rates in July and low HBRs could be explained by a shift in the age structure of the mosquito populations in this site. For example, if for some reason larval mortality was high for a period, this would result in fewer adult mosquitoes being collected, but they would be older and thus have a higher sporozoite rate.

Somewhat puzzling, Cano *et al.*[23] conducted malaria mosquito collections in 1998–99 on Bioko Island but did not identify any *An. melas* specimens, although this species was found at considerable frequency in the current collections in both Riaba and Arena Blanca. *Anopheles melas* was also present in Riaba in the 2003–2004 pre-spray BIMCP collections with very high sporozoite rates [3]. Possibly, most of the collection sites of Cano *et al.*[23] were located further inland, away from the coast where *An. melas* breeding sites are located.

In Arena Blanca, *An. melas* was the major malaria vector. This site is close to the town of Luba, but the environmental and socioeconomic conditions differ markedly from Luba. Arena Blanca is a small fishing village comprised of approximately 50 houses located directly on the beach. The majority of people living there are Annobonese fishermen with a culture distinctly different from the rest of the island. Arena Blanca is also a popular weekend beach resort for people from around the island. Although the calculated EIR is the lowest on the island, people, including weekenders, are still exposed to an average of about 22 outdoor biting malaria mosquitoes during the first half of the night, of which ~1% might be infected.

Our results indicate that the S form of *An. gambiae* s.s. was eliminated from the island while the M form remains. Prior to the BIMCP, 36% of the *An. gambiae* s.s. mosquitoes collected on Bioko consisted of the M form, but by the end of the third spray round this had increased to 80% [6]. Successive analyses showed that the M-form increased steadily from 80% in 2005 (n = 189), 98% in 2006 (n = 467), 94% in 2007 (n = 79), and 100% in 2008 (n = 149) (BIMCP unpublished data). In the 2009 collections, none of the 3,043 mosquitoes analyzed (consisting of a proportional sample across all sites and time points) belonged to the S form of *An. gambiae* s.s. A possible explanation for the apparent disappearance of the S form could be differences in the frequencies of *kdr* mutation between the two molecular forms. Knockdown resistance allele frequencies among *An. gambiae* on Bioko Island are unusual in that the L1014F allele was at high frequency in the M form, rather than the S form early on [28]. This contrasts with the situation on the West African mainland, where this allele first spread through S form populations, prior to introgression into the M form [29]. This could potentially have led to the persistence of the M form and the disappearance of the S form on Bioko.

On the other hand, a recent modeling study [Kiszewski *et al.* in review] indicated that *kdr* alleles limit the efficacy of IRS only to a certain degree making it hard to see how this difference in *kdr* frequency resulted in the disappearance of the S form. Likewise, this does not explain the continued decline of the S form after the BIMCP switched to using carbamates in IRS instead of pyrethroids. In the equatorial rain forest of Central Africa, the M and S forms are widely sympatric and no niche differentiation has been detected [30]. Another possibility could be that the M and S forms differed in their propensity to feed and/or rest indoors, making the M form less susceptible to IRS and LLINs, although an earlier rather limited survey found no evidence of outdoor feeding *An. gambiae* s.l. on Bioko Island [18]. In addition, or alternatively, the M form may have been less susceptible to the two classes of insecticides used in the IRS and LLIN campaign due to *kdr* and more efficient detoxification.

The L1014F mutation was the only insecticide resistance allele detected among the *An. gambiae* mosquitoes on Bioko. The frequency of L1014F allele varied but exceeded 40% in most locations. The highest frequency was observed in the urban populated areas in the north of the island; reaching nearly 90% in Semu. This suggests that insecticide use from previous and present pyrethroid-based control campaigns, but most likely household insecticide use as well, has resulted in increased frequency of the L1014F allele*.* Large businesses and companies often have their own mosquito control programs independent from the BIMCP, further reinforcing selection pressure for insecticide resistance. Agricultural use of insecticides is not common in Equatorial Guinea [31,32] and is therefore unlikely to impact *kdr* allele frequencies.

Following an initial spray round with a pyrethroid, the BIMCP switched to bendiocarb, a carbamate insecticide, in the IRS. This decision was based on the presence of *kdr* and absence of *ace-1*^*R*^ mutations. As the frequency of *kdr* mutation was low in *An. gambiae* s.s. in Riaba and the mutation was absent in *An. melas* in Arena Blanca, these locations are favorable for a change in insecticide used in IRS. The NMCP of Equatorial Guinea has recently adopted the WHO Guidelines for Insecticide Resistance Management (GPIRM) [33] and decided to switch to pyrethroids on a rotational basis for the whole of Bioko Island.

Although four years of control on Bioko was successful in greatly reducing child mortality [3] and having a dramatic impact on the effective population size of *An. gambiae* and *An. melas*[34] entomological indicators indicate that, even if the force of transmission has been substantially reduced relative to pre-intervention levels, it still remains high on the island relative to other African contexts, and that there remain foci of very high transmission. As the current twice-a-year spray rounds with bendiocarb have insufficient residual duration to provide effective control on Bioko [27], the IRS intervention needs to be supported by much higher LLIN coverage and supplemented with an additional set of focally administered control measures [5]. The BIMCP is currently piloting such a stratified focal approach in Punta Europa, combining IRS with universal LLIN distribution, Focal Mass Screening and Treatment (FMST), reinforced IEC/BCC and source reduction through larval source management. Future focal efforts might also evaluate the efficacy of infrastructural modifications and environmental management, along with testing and possible introduction of other transmission barriers (e.g. insecticide treated materials) and personal protection methods. Collaboration between BIMCP, the airport administration, and local businesses is also a strategy that should be explored to reduce mosquito breeding in these areas through infrastructural modification and environmental management. Although the precise impact of outdoor biting on malaria prevalence still needs to be fully clarified, the fact that extensive outdoor biting is occurring on Bioko Island [8] and other parts of Africa [35] suggests that alternative and innovative control options should be developed and implemented to address this problem.

## Conclusions

Results from this study show that in spite of the concerted vector control efforts of the National Malaria Control Program with support from the Bioko Island Malaria Control Project (BIMCP) since 2004, and in spite of the general reduction in the force of transmission, parasite prevalence and mortality on the island, a number of persistent foci of high transmission remain on the island. Principal among these is the Punta Europa area in northwestern Bioko Island, where the entomological inoculation rates remain among the highest ever recorded. People in this area are still exposed to more than 800 infective mosquito bites per year with potentially higher outdoor rates. The area seems to be favorable for malaria mosquito breeding, possibly due to the flat and open areas around the international airport. The number of *Anopheles* mosquitoes landing on humans and attempting to feed is also among the highest recorded. The twice yearly indoor residual sprays of the BIMCP have not been sufficient to control the density of infected mosquitoes biting people in this area. The recent efforts in Punta Europa to supplement IRS with full coverage of LLINs, Focal Mass Screening and Treatment (FMST), reinforced IEC/BCC and community-based larval source management on a focal basis is an appropriate response to the prevailing transmission characteristics. Depending on the outcome of this pilot initiative, consideration should also be given to coordinated infrastructural modification and environmental management with the airport authorities and local industries. These added efforts might help to further reduce mosquito breeding and potentially also outdoor biting. In addition, the BIMCP may also want to contemplate the introduction of other transmission barriers and personal protection methods in the future. As Bioko Island, like more and more malarious countries, aims for malaria elimination, control strategies will likely need to be stratified to reflect heterogeneous localized transmission characteristics. Elimination of malaria in this setting may require the introduction of focal strategies to successfully address transmission hot spots, including those attributable to outdoor biting.

## Competing interests

The authors declare that they have no competing interests.

## Authors' contributions

HJO: Conceived, supervised, and planned the study, supervised and performed field collections. Wrote the first draft manuscript. VPR: Performed molecular analyses. SA: Participated in the study design, supervised and participated in field collections, and provided editorial input. AM: Participated in field collections. Provided editorial input. MRR: Participated in study design and provided editorial input. VK: Performed molecular analyses. CS: Participated in study design and provided editorial input. LS: Participated in study design and provided editorial input. IK: Participated in study design and provided editorial input. MAS: Participated in study design, supervised the molecular analysis, and contributed to manuscript preparation. All authors read and approved the final version of the manuscript.
